# Supplementary material for: How does framing influence preference for multiple solutions to societal problems?
Source: PLoS One. 2023 May 17;18(5):e0285793. doi: 10.1371/journal.pone.0285793 (PMC10191302; doi:10.1371/journal.pone.0285793)
Supplement: S1 File — (DOCX) [file pone.0285793.s001.docx]

**Supporting Information**

**S1. Eight environmental and social problems presented in the four conditions**

**1. Climate change**

1. **Control frame:** Climate change, in its current usage, refers to rising temperatures and changing weather patterns driven by the emission of greenhouse gases (e.g., carbon dioxide). Climate change is a serious global crisis, causing extreme weather events (e.g., heavy rainfalls, heat waves, stronger hurricanes and snowstorms) which can damage personal health and safety, food production, economic prosperity, and many other aspects of society. Therefore, urgent action is needed to reduce emissions and mitigate the impact of extreme weather.

   Multiple facts are known about this complex issue. For example:
   - **Global temperatures** have risen 1.1 °C (1.9 °F) over the past century.
   - **Land temperatures** are rising at a faster rate than ocean temperatures, because the capacity of land to absorb heat is smaller than oceans.
   - **Greenhouse gases** in the atmosphere absorb and reemit radiation, which warms up the Earth’s surface.
   - **Carbon dioxide and methane** are common greenhouse gases. Atmospheric carbon dioxide concentration has increased by 40% over the past century.
2. **Multi-cause frame:** Climate change, in its current usage, refers to rising temperatures and changing weather patterns driven by the emission of greenhouse gases (e.g., carbon dioxide). Climate change is a serious global crisis, causing extreme weather events (e.g., heavy rainfalls, heat waves, stronger hurricanes and snowstorms) which can damage personal health and safety, food production, economic prosperity, and many other aspects of society. Therefore, urgent action is needed to reduce emissions and mitigate the impact of extreme weather.

   Multiple causes contribute to this complex issue. For example:
   - **Electricity** is largely generated from the burning of fossil fuels (coal, oil, and natural gas), which results in significant greenhouse gas emissions.
   - **Deforestation** releases carbon stored in plants into the environment, and reduces the ability of forests to absorb carbon.
   - **Transportation** produces a large amount of greenhouse gases because most vehicles (e.g., cars, ships, planes) run on fossil fuels.
   - **Producing food** is often a high-emission process. For example, livestock farming generates methane.
3. **Multi-impact frame:** Climate change, in its current usage, refers to rising temperatures and changing weather patterns driven by the emission of greenhouse gases (e.g., carbon dioxide). Climate change is a serious global crisis, causing extreme weather events (e.g., heavy rainfalls, heat waves, stronger hurricanes and snowstorms) which can damage personal health and safety, food production, economic prosperity, and many other aspects of society. Therefore, urgent action is needed to reduce emissions and mitigate the impact of extreme weather.

   Multiple impacts result from this complex issue. For example:
   - **Personal safety** is at risk from climate disasters, such as property damage from hurricanes and floods, and afflictions and death from extreme heat.
   - **Diseases** spread more quickly and expand their range under higher temperatures, which could lead to more severe outbreaks.
   - **Food shortages** become more common due to droughts and pest infestations, which reduce crop production.
   - **Income inequality** increases partly because the impact of climate change is disproportionately more severe in poorer communities.
4. **Multi-solution frame:** Climate change, in its current usage, refers to rising temperatures and changing weather patterns driven by the emission of greenhouse gases (e.g., carbon dioxide). Climate change is a serious global crisis, causing extreme weather events (e.g., heavy rainfalls, heat waves, stronger hurricanes and snowstorms) which can damage personal health and safety, food production, economic prosperity, and many other aspects of society. Therefore, urgent action is needed to reduce emissions and mitigate the impact of extreme weather.

   Multiple solutions are needed to address this complex issue. For example:
   - **Governments** should enact climate policies, set up carbon markets and taxes, or provide subsidies to sustainable industries, to curtail emissions.
   - **Individuals** should take actions to reduce personal emissions, such as driving less, flying less, or eating less red meat.
   - **Businesses** should develop more affordable climate-friendly technology, such as electric cars and renewable energy.
   - **Non-governmental organizations** should advocate governments, individuals, and businesses to make substantial changes in the system to reduce emissions.

How severe a problem do you think climate change is?

1=Not severe at all, 2=Slightly severe, 3=Somewhat severe, 4=Moderately severe, 5=Considerably severe, 6=Very severe, 7=Extremely severe

How urgent a problem do you think climate change is?

1=Not urgent at all, 2=Slightly urgent, 3=Somewhat urgent, 4=Moderately urgent, 5=Considerably urgent, 6=Very urgent, 7=Extremely urgent

Please select which of the following you think is a good solution to climate change.

1. Government action (e.g., enact climate policies, set carbon markets and taxes, provide subsidies to sustainable industries)
2. Individual action (e.g., drive less, fly less, eat less meat)
3. Multiple solutions are needed in addition to 1 and 2
4. Neither 1 nor 2

**2. Crop yield and biodiversity**

1. **Control frame:** Increasing crop yield is an essential part of feeding the world. This is achieved partly through expansion (turning natural habitats into agricultural lands) and intensification (increasing productivity through pesticides, fertilizers). These practices can lead to biodiversity loss (e.g., reduced animal and plant population), which could have a negative impact on crop yield (e.g., reduced pollination from bees). Therefore, urgent action is needed to attain a better balance between agriculture and biodiversity.

   Multiple facts are known about this complex issue. For example:
   - **Agricultural expansion** means that natural habits are converted into agricultural lands across the world, especially in tropical regions, to meet the demands of a growing human population.
   - **Agricultural intensification** refers to improvements that increase production per unit of land. Examples include the use of pesticides and fertilizers, irrigation, labor input, and genetic modification of crops.
   - **Biodiversity** has many benefits for crops. Pollinators (e.g., bees) help pollinate crops, while predators (e.g., carnivorous beetles) help suppress pests.
2. **Multi-cause frame:** Increasing crop yield is an essential part of feeding the world. This is achieved partly through expansion (turning natural habitats into agricultural lands) and intensification (increasing productivity through pesticides, fertilizers). These practices can lead to biodiversity loss (e.g., reduced animal and plant population), which could have a negative impact on crop yield (e.g., reduced pollination from bees). Therefore, urgent action is needed to attain a better balance between agriculture and biodiversity.

   Multiple causes contribute to this complex issue. For example:
   - **Growing global demand** for food is driving agricultural expansion and intensification, which degrade natural habitats at a rapid pace.
   - **Industrial farming** favors large monocultures (croplands with only one type of crop). This practice has created large swaths of homogenous land with low biodiversity.
   - **Climate change** has resulted in the deterioration of croplands, particularly in developing countries. To make up for production loss, farmers often resort to more unsustainable practices (e.g., constant sowing without letting the land recover).
3. **Multi-impact frame:** Increasing crop yield is an essential part of feeding the world. This is achieved partly through expansion (turning natural habitats into agricultural lands) and intensification (increasing productivity through pesticides, fertilizers). These practices can lead to biodiversity loss (e.g., reduced animal and plant population), which could have a negative impact on crop yield (e.g., reduced pollination from bees). Therefore, urgent action is needed to attain a better balance between agriculture and biodiversity.

   Multiple impacts result from this complex issue. For example:
   - **Ecosystem services** that are important to crop yield (e.g., pollination, natural predators of agricultural pests) are disappearing in some places.
   - **Land degradation**, which is often associated with permanent loss of productivity, has occurred in some regions due to unsustainable agriculture. These degraded lands lack the capacity to support agriculture and biodiversity.
   - **Imbalances in soil nutrients** (nitrogen, phosphorus) can be partially attributed to unsustainable agriculture. These changes can decrease the nutritional value of crops and jeopardize food safety.
4. **Multi-solution frame:** Increasing crop yield is an essential part of feeding the world. This is achieved partly through expansion (turning natural habitats into agricultural lands) and intensification (increasing productivity through pesticides, fertilizers). These practices can lead to biodiversity loss (e.g., reduced animal and plant population), which could have a negative impact on crop yield (e.g., reduced pollination from bees). Therefore, urgent action is needed to attain a better balance between agriculture and biodiversity.

   Multiple solutions are needed to address this complex issue. For example:
   - **Land sparing**, which involves making agriculture more intensive and high-yielding, is needed to achieve high production over a smaller area of land, while leaving the surrounding natural habitat untouched.
   - **Land sharing**, which involves more wildlife-friendly but less intense (i.e., lower-yielding) agriculture over a larger area of land, is needed to allow free movement of wildlife.
   - **Integration of non-crop plants** into farmlands (e.g., hedgerows) should be employed to support the biodiversity that benefits crop yield.

How severe a problem do you think the imbalance between crop yield and biodiversity is?

1=Not severe at all, 2=Slightly severe, 3=Somewhat severe, 4=Moderately severe, 5=Considerably severe, 6=Very severe, 7=Extremely severe

How urgent a problem do you think the imbalance between crop yield and biodiversity is?

1=Not urgent at all, 2=Slightly urgent, 3=Somewhat urgent, 4=Moderately urgent, 5=Considerably urgent, 6=Very urgent, 7=Extremely urgent

Please select which of the following you think is a good solution to the imbalance between crop yield and biodiversity.

1. Land sparing (intense, high-yielding agriculture over a small area of land; leaves the surrounding natural habitat untouched)
2. Land sharing (more wildlife-friendly but less intense and low-yielding agriculture over a larger area of land)
3. Multiple solutions are needed in addition to 1 and 2
4. Neither 1 nor 2

**3. Food waste**

1. **Control frame:** Globally about a third of all food produced ends up as waste. Food waste occurs in all stages of the food cycle, from production to transportation to consumption. Food waste is not only a waste of resources that could have gone to food-insecure households, but can also produce greenhouse gas (e.g., methane), and cause air and water pollution. Food waste is therefore a serious socioeconomic and environmental issue, and urgent action is needed to reduce food waste.

   Multiple facts are known about this complex issue. For example:
   - **The food industry** is an amalgamation of several sectors, including production (e.g., agriculture), manufacturing, processing, packaging, marketing, distribution, and retailing. Food waste occurs in all these sectors.
   - **Large, industrialized food companies** produce most of the food consumed in Western countries. However, there are still many traditional, family-run food businesses.
   - **Many countries in the world** produce a significant amount of food waste. Most of this food waste consists of fruits and vegetables.
2. **Multi-cause frame:** Globally about a third of all food produced ends up as waste. Food waste occurs in all stages of the food cycle, from production to transportation to consumption. Food waste is not only a waste of resources that could have gone to food-insecure households, but can also produce greenhouse gas (e.g., methane), and cause air and water pollution. Food waste is therefore a serious socioeconomic and environmental issue, and urgent action is needed to reduce food waste.

   Multiple causes contribute to this complex issue. For example:
   - **Ugly produce** (fruits and vegetables that are safe to eat but fail to meet cosmetic standards) is not harvested and discarded. This results in significant food waste in the production stage.
   - **Expiration dates** are often arbitrary or not well-labeled. This creates confusion among both consumers and retailers, and results in large amounts of edible food being thrown away.
   - **Consumers** often buy more food than they can consume, resulting in significant household food waste.
3. **Multi-impact frame:** Globally about a third of all food produced ends up as waste. Food waste occurs in all stages of the food cycle, from production to transportation to consumption. Food waste is not only a waste of resources that could have gone to food-insecure households, but can also produce greenhouse gas (e.g., methane), and cause air and water pollution. Food waste is therefore a serious socioeconomic and environmental issue, and urgent action is needed to reduce food waste.

   Multiple impacts result from this complex issue. For example:
   - **Valuable water and energy resources** are wasted as a result of food waste, since these resources are important for food production, packaging, and transportation.
   - **Food inequality** is associated with food waste, where lower income individuals are not getting enough food, while higher income individuals are overconsuming food.
   - **Certain environmental issues** are closely linked to food waste. Decomposing food waste in landfills generates a significant amount of greenhouse gases, which contribute to global warming.
4. **Multi-solution frame:** Globally about a third of all food produced ends up as waste. Food waste occurs in all stages of the food cycle, from production to transportation to consumption. Food waste is not only a waste of resources that could have gone to food-insecure households, but can also produce greenhouse gas (e.g., methane), and cause air and water pollution. Food waste is therefore a serious socioeconomic and environmental issue, and urgent action is needed to reduce food waste.

   Multiple solutions are needed to address this complex issue. For example:
   - **The food industry** should shift to more sustainable practices such as avoiding supersizing packages and portions, reducing aesthetical standards for produce, and donating surplus food to charities and food-insecure communities.
   - **Consumers** should manage their food carefully by avoiding overbuying, managing food storage, eating leftovers, and composting food waste.
   - **Governments** should enact policies to distribute food more evenly, manage food waste, and create incentives for the food industry to be less wasteful.

How severe a problem do you think food waste is?

1=Not severe at all, 2=Slightly severe, 3=Somewhat severe, 4=Moderately severe, 5=Considerably severe, 6=Very severe, 7=Extremely severe

How urgent a problem do you think food waste is?

1=Not urgent at all, 2=Slightly urgent, 3=Somewhat urgent, 4=Moderately urgent, 5=Considerably urgent, 6=Very urgent, 7=Extremely urgent

Please select which of the following you think is a good solution to food waste.

1. Food industry action (e.g., avoid supersizing packages and portions, reduce aesthetical standards for produce, donate surplus food)
2. Consumer action (e.g., avoid overbuying, manage food storage, eat leftovers, compost food waste)
3. Multiple solutions are needed in addition to 1 and 2
4. Neither 1 nor 2

**4. Plastic pollution**

1. **Control frame:** Plastic pollution occurs when plastic waste (e.g., plastic bags, containers, cutleries) enters the environment. Being slow to decompose, plastic can accumulate both on land and in water. In its solid form, plastic can damage infrastructure (e.g., clog sewage drains). Furthermore, when plastic particles enter rivers and oceans, they often end up being ingested by animals and ultimately humans. Plastic pollution is therefore a serious health and environmental issue, and urgent action is needed to reduce plastic pollution.

   Multiple facts are known about this complex issue. For example:
   - **Raw materials** used to make most plastics are from fossil fuels (oil, natural gas). Some plastics use plant-derived materials, which unlike regular plastic, are decomposable by living organisms.
   - **Packaging** accounts for about 40% of all plastic waste, followed by building applications (e.g., piping) and textiles.
   - **Plastic is generally not degradable** and accumulates in the environment. Plastic waste has been accumulating steadily since the 1950s, when plastic was first mass produced.
2. **Multi-cause frame:** Plastic pollution occurs when plastic waste (e.g., plastic bags, containers, cutleries) enters the environment. Being slow to decompose, plastic can accumulate both on land and in water. In its solid form, plastic can damage infrastructure (e.g., clog sewage drains). Furthermore, when plastic particles enter rivers and oceans, they often end up being ingested by animals and ultimately humans. Plastic pollution is therefore a serious health and environmental issue, and urgent action is needed to reduce plastic pollution.

   Multiple causes contribute to this complex issue. For example:
   - **A variety of consumer products**, ranging from containers to bags to single-use utensils, contain plastic. The overuse of plastic results in its accumulation.
   - **Textiles, tires, and dust** are the main sources of plastic particles. Due to their small size, plastic particles are easily carried by air and water, and have a widespread presence.
   - **Fishing nets** are a major source of marine plastic pollution, and can circulate around the globe via ocean currents.
3. **Multi-impact frame:** Plastic pollution occurs when plastic waste (e.g., plastic bags, containers, cutleries) enters the environment. Being slow to decompose, plastic can accumulate both on land and in water. In its solid form, plastic can damage infrastructure (e.g., clog sewage drains). Furthermore, when plastic particles enter rivers and oceans, they often end up being ingested by animals and ultimately humans. Plastic pollution is therefore a serious health and environmental issue, and urgent action is needed to reduce plastic pollution.

   Multiple impacts result from this complex issue. For example:
   - **Health problems** arise from waterborne plastic particles, which can be easily absorbed by the human body and may accumulate in the brain, liver, and other organs.
   - **Plastic debris** accumulates in large amounts in croplands, freshwater, and other terrestrial environments, which can cause problems for food safety.
   - **Marine wildlife** is severely affected by plastic pollution. Marine animals can get entangled in plastic nets and rings, or mistake plastics for food (e.g., turtles).
4. **Multi-solution frame:** Plastic pollution occurs when plastic waste (e.g., plastic bags, containers, cutleries) enters the environment. Being slow to decompose, plastic can accumulate both on land and in water. In its solid form, plastic can damage infrastructure (e.g., clog sewage drains). Furthermore, when plastic particles enter rivers and oceans, they often end up being ingested by animals and ultimately humans. Plastic pollution is therefore a serious health and environmental issue, and urgent action is needed to reduce plastic pollution.

   Multiple solutions are needed to address this complex issue. For example:
   - **Plastic consumption should be reduced**, especially single-use plastics (e.g., utensils, plastic bags, wrapping). Alternatives such as paper and glass should be adopted. In cases where plastic is necessary, biodegradable plastic should be used.
   - **More effective management of plastic waste** is needed, such as incineration, recycling, or disposal in well-managed landfills.
   - **The environment should be well-monitored** for plastic contamination, especially water supplies in the vicinity of residential or agricultural areas.

How severe a problem do you think plastic pollution is?

1=Not severe at all, 2=Slightly severe, 3=Somewhat severe, 4=Moderately severe, 5=Considerably severe, 6=Very severe, 7=Extremely severe

How urgent a problem do you think plastic pollution is?

1=Not urgent at all, 2=Slightly urgent, 3=Somewhat urgent, 4=Moderately urgent, 5=Considerably urgent, 6=Very urgent, 7=Extremely urgent

Please select which of the following you think is a good solution to plastic pollution.

1. Reduce plastic consumption and adopt alternatives such as paper, glass, and biodegradable materials
2. Manage plastic waste more effectively (e.g., incineration, recycling, disposal in well-managed landfills)
3. Multiple solutions are needed in addition to 1 and 2
4. Neither 1 nor 2

**Attention check**

About one-fifth of the world population lives in earthquake-prone areas, and the global population of earthquake-prone areas is growing at a faster rate than the global population. Earthquakes result in significant casualties, damage to buildings and infrastructure, and secondary disasters (e.g., landslides, tsunamis) every year. This is an attention check. Please choose option 3 below to show that you are paying attention and to receive payment from this study. After choosing option 3, please proceed to the next question. Because of this, earthquake hazard mitigation is a serious issue for seismically active countries.

Please select which of the following you think is a good solution to earthquake hazard mitigation.

1. Government action (e.g., enforce earthquake-proof building codes, design more resistant infrastructure)
2. Individual action (e.g., prepare an emergency kit, secure furniture, and learn how to turn off gas, electricity and water in homes)
3. Multiple solutions are needed in addition to 1 and 2
4. Neither 1 nor 2

**5. Homelessness**

1. **Control frame:** Homelessness is the state of lacking safe and stable housing. Approximately 2% of the world population is currently homeless. Moreover, in some countries, including the U.S., homelessness has been exacerbated by evictions during the COVID-19 pandemic. Homelessness leads to a number of negative outcomes, such as reduced life expectancy, substance abuse, mental health issues, lack of social connections, and victimization by violent crime. Therefore, homelessness is a serious social issue that needs to be addressed with urgency.

   Multiple facts are known about this complex issue. For example:
   - **Around 150 million people** worldwide are currently homeless. In the U.S. alone, an estimated 0.5 million people are currently homeless.
   - **Men** represent about 60% of the homeless population in the U.S. Half of the homeless population are racial minorities. In addition, many homeless individuals have physical or mental disabilities.
   - **The life expectancy** of people experiencing homelessness is about 8 to 13 years shorter than that of the general population.
2. **Multi-cause frame:** Homelessness is the state of lacking safe and stable housing. Approximately 2% of the world population is currently homeless. Moreover, in some countries, including the U.S., homelessness has been exacerbated by evictions during the COVID-19 pandemic. Homelessness leads to a number of negative outcomes, such as reduced life expectancy, substance abuse, mental health issues, lack of social connections, and victimization by violent crime. Therefore, homelessness is a serious social issue that needs to be addressed with urgency.

   Multiple causes contribute to this complex issue. For example:
   - **Financial problems** (e.g., the lack of income, income insecurity) are the primary drivers of homelessness. Financial problems are exacerbated by economic problems, such as inflation and the pandemic.
   - **Lack of support** from family and friends can cause people to lose social connections and social capital, contributing to homelessness. This is especially true for people with mental health challenges.
   - **Lack of affordable housing** can cause people to lose stable housing and become homeless.
3. **Multi-impact frame:** Homelessness is the state of lacking safe and stable housing. Approximately 2% of the world population is currently homeless. Moreover, in some countries, including the U.S., homelessness has been exacerbated by evictions during the COVID-19 pandemic. Homelessness leads to a number of negative outcomes, such as reduced life expectancy, substance abuse, mental health issues, lack of social connections, and victimization by violent crime. Therefore, homelessness is a serious social issue that needs to be addressed with urgency.

   Multiple impacts result from this complex issue. For example:
   - **Wellbeing** is reduced by homelessness, and is associated with a number of physical and mental health problems. Homeless children are especially susceptible, which can lead to intergenerational poverty.
   - **Violence and crime** often increase with homelessness. Homeless individuals suffer from increased assaults, thefts, and trauma. This can shorten their lifespan by 10 years.
   - **Economic costs** of funding anti-homeless programs and dealing with the accompanying issues of homelessness (e.g., law enforcement, healthcare) are huge.
4. **Multi-solution frame:** Homelessness is the state of lacking safe and stable housing. Approximately 2% of the world population is currently homeless. Moreover, in some countries, including the U.S., homelessness has been exacerbated by evictions during the COVID-19 pandemic. Homelessness leads to a number of negative outcomes, such as reduced life expectancy, substance abuse, mental health issues, lack of social connections, and victimization by violent crime. Therefore, homelessness is a serious social issue that needs to be addressed with urgency.

   Multiple solutions are needed to address this complex issue. For example:
   - **Social and health services**, such as emergency shelters, food stamps, medical services (e.g., detox centers), and housing support, should be readily accessible to individuals experiencing homeless.
   - **Cash transfers** should be provided, which is a direct cash payment to individuals experiencing homelessness so they can afford necessities (e.g., rent, furniture, food).
   - **Systematic changes**, such as healthcare reform, building more affordable homes, and welfare service reform are needed to reduce homelessness.

How severe a problem do you think homelessness is?

1=Not severe at all, 2=Slightly severe, 3=Somewhat severe, 4=Moderately severe, 5=Considerably severe, 6=Very severe, 7=Extremely severe

How urgent a problem do you think homelessness is?

1=Not urgent at all, 2=Slightly urgent, 3=Somewhat urgent, 4=Moderately urgent, 5=Considerably urgent, 6=Very urgent, 7=Extremely urgent

Please select which of the following you think is a good solution to homelessness.

1. Social and health services (e.g., emergency shelters, food stamps, medical services, housing support)
2. Cash transfers (direct cash payment to individuals experiencing homelessness)
3. Multiple solutions are needed in addition to 1 and 2
4. Neither 1 nor 2

**6. Police reform**

1. **Control frame:** The U.S. police force has come under intense scrutiny in recent years for a variety of issues. For example, confrontations with the police routinely lead to the excessive use of force or even death, especially when the other party is a racial minority. Meanwhile, police unions often wield considerable power over local politics, and there is a general lack of police accountability. These problems mean that the current police force is in urgent need of reform.

   Multiple facts are known about this complex issue. For example:
   - **The main function** of the police force is to investigate criminal activity, refer investigation results to prosecutors, and temporarily detain suspects.
   - **Police unions** are trade unions representing the police force. About 80% of police officers belong to a police union. Police unions are often seen as an impediment to criminal justice reform.
   - **Police-related deaths** in the U.S. are around 1,000 people a year, which is significantly higher than other developed countries.
2. **Multi-cause frame:** The U.S. police force has come under intense scrutiny in recent years for a variety of issues. For example, confrontations with the police routinely lead to the excessive use of force or even death, especially when the other party is a racial minority. Meanwhile, police unions often wield considerable power over local politics, and there is a general lack of police accountability. These problems mean that the current police force is in urgent need of reform.

   Multiple causes contribute to this complex issue. For example:
   - **Police unions** have considerable power, allowing police misconduct to go unchecked and blocking reform proposals. Police unions can also leverage their influence on local communities by threatening to reduce policing.
   - **Police training** is centered too much around an us-versus-them mentality, which increases the likelihood of police to resort to deadly force.
   - **Institutional racism** has caused police to target individuals based on their race instead of their behavior, resulting in disproportionate harm to racial minorities.
3. **Multi-impact frame:** The U.S. police force has come under intense scrutiny in recent years for a variety of issues. For example, confrontations with the police routinely lead to the excessive use of force or even death, especially when the other party is a racial minority. Meanwhile, police unions often wield considerable power over local politics, and there is a general lack of police accountability. These problems mean that the current police force is in urgent need of reform.

   Multiple impacts result from this complex issue. For example:
   - **Racial tensions** tend to be exacerbated by police brutality, since most victims of police brutality are members of racial minorities.
   - **The lack of financial compensation**, due to insufficient police accountability, makes it more difficult for families affected by police misconduct to recover (from injuries, property damage, deaths).
   - **Police militarization** (i.e., police using military equipment), combined with the lack of proper training, can make confrontations with the police deadlier, and further reduce police accountability.
4. **Multi-solution frame:** The U.S. police force has come under intense scrutiny in recent years for a variety of issues. For example, confrontations with the police routinely lead to the excessive use of force or even death, especially when the other party is a racial minority. Meanwhile, police unions often wield considerable power over local politics, and there is a general lack of police accountability. These problems mean that the current police force is in urgent need of reform.

   Multiple solutions are needed to address this complex issue. For example:
   - **Internal reform**, which includes improving police training and accountability, restricting the use of deadly force, and reining in police unions is necessary.
   - **Reallocation** of a portion of government funding for police departments to social programs that could reduce the likelihood of crime (e.g., anti-poverty programs, mental health programs) should be considered.
   - **Local organizations** (e.g., neighborhood watches, restorative justice teams) should take up a more active role in safeguarding their community.

How severe a problem do you think police reform is?

1=Not severe at all, 2=Slightly severe, 3=Somewhat severe, 4=Moderately severe, 5=Considerably severe, 6=Very severe, 7=Extremely severe

How urgent a problem do you think police reform is?

1=Not urgent at all, 2=Slightly urgent, 3=Somewhat urgent, 4=Moderately urgent, 5=Considerably urgent, 6=Very urgent, 7=Extremely urgent

Please select which of the following you think is a good solution to police reform.

1. Internal reform (e.g., improve police training and accountability, restrict the use of excessive force, rein in police unions)
2. Reallocation of police funding to social programs that could reduce the likelihood of crime (e.g., anti-poverty programs, mental health programs)
3. Multiple solutions are needed in addition to 1 and 2
4. Neither 1 nor 2

**7. Public education**

1. **Control frame:** The public education system of the U.S. is often criticized for low student academic performance. The average performance of U.S. K-12 students in areas of science, mathematics, and reading has remained stagnant over the past two decades, and currently lags behind many developed countries. Furthermore, the performance of U.S. students shows a progressive decline in proficiency with age in some subjects (e.g., mathematics). Because of these issues, the U.S. public education system is in urgent need of improvement.

   Multiple facts are known about this complex issue. For example:
   - **The majority** of U.S. K-12 students (87%) attend public schools. The rest attend private schools or are home-schooled.
   - **Student to teacher ratio** has remained around 16 for the last two decades. There is significant disparity between states.
   - **Education spending per student** in the U.S. is one of the highest in the world.
   - **The math skills** of U.S. K-12 students typically rank around 30th globally.
2. **Multi-cause frame:** The public education system of the U.S. is often criticized for low student academic performance. The average performance of U.S. K-12 students in areas of science, mathematics, and reading has remained stagnant over the past two decades, and currently lags behind many developed countries. Furthermore, the performance of U.S. students shows a progressive decline in proficiency with age in some subjects (e.g., mathematics). Because of these issues, the U.S. public education system is in urgent need of improvement.

   Multiple causes contribute to this complex issue. For example:
   - **Teaching quality** is unsatisfactory. This is due to a multitude of factors, from outdated teaching methods (e.g., teacher-student interactions) to poor training.
   - **Students from disadvantaged communities** are not getting enough support, preventing them from keeping up with their peers.
   - **Overcrowding and teacher shortages** are a problem in some schools, meaning some students are not receiving enough attention.
   - **Education spending and teacher pay** have remained stagnant for years.
3. **Multi-impact frame:** The public education system of the U.S. is often criticized for low student academic performance. The average performance of U.S. K-12 students in areas of science, mathematics, and reading has remained stagnant over the past two decades, and currently lags behind many developed countries. Furthermore, the performance of U.S. students shows a progressive decline in proficiency with age in some subjects (e.g., mathematics). Because of these issues, the U.S. public education system is in urgent need of improvement.

   Multiple impacts result from this complex issue. For example:
   - **Student wellbeing** is often negatively impacted by poor academic performance.
   - **Students from disadvantaged communities** are finding it difficult to keep up with their peers, which further exacerbates inequality.
   - **Standardized testing**, meant to keep track of and boost student performance, have not been effective and can be an unnecessary burden.
   - **Art and music**, which many children enjoy, have been set aside in favor of more focus on core subjects.
4. **Multi-solution frame:** The public education system of the U.S. is often criticized for low student academic performance. The average performance of U.S. K-12 students in areas of science, mathematics, and reading has remained stagnant over the past two decades, and currently lags behind many developed countries. Furthermore, the performance of U.S. students shows a progressive decline in proficiency with age in some subjects (e.g., mathematics). Because of these issues, the U.S. public education system is in urgent need of improvement.

   Multiple solutions are needed to address this complex issue. For example:
   - **Teaching quality** should be improved by incorporating more modern teaching methods, raising teacher pay, and increasing curriculum flexibility.
   - **Student wellbeing** should be prioritized so students can focus better on studying. Schools should offer better counselling services, prevent bullying at school, and provide better support to children from lower-income households.
   - **Parents** should be more involved as a mediator between students and schools.
   - **Governments** should increase education spending.

How severe a problem do you think low student academic performance in public schools is?

1=Not severe at all, 2=Slightly severe, 3=Somewhat severe, 4=Moderately severe, 5=Considerably severe, 6=Very severe, 7=Extremely severe

How urgent a problem do you think low student academic performance in public schools is?

1=Not urgent at all, 2=Slightly urgent, 3=Somewhat urgent, 4=Moderately urgent, 5=Considerably urgent, 6=Very urgent, 7=Extremely urgent

Please select which of the following you think is a good solution to low student academic performance in public schools.

1. Improve teaching quality (e.g., incorporate more modern teaching methods, raise teacher pay, increase curriculum flexibility)
2. Improve student wellbeing so they can focus better on studying (e.g., offer better counselling services, prevent bullying at school, provide better support to children from lower-income households)
3. Multiple solutions are needed in addition to 1 and 2
4. Neither 1 nor 2

**8. Early pandemic response**

1. **Control frame:** The impact of the ongoing COVID-19 pandemic can still be felt across much of the globe. The pandemic has resulted in a large number of casualties, as well as significant socioeconomic disruption, ranging from unemployment to evictions to economic recession. Moreover, studies show that large-scale disease outbreaks will become increasingly frequent in the future. In order to minimize the impact of future pandemics, it is urgent to come up with an effective early response system.

   Multiple facts are known about this complex issue. For example:
   - **Several pandemics** have occurred in the past century. Notable recent pandemics include SARS, H1N1, Ebola, and the Zika virus.
   - **The 1918 influenza pandemic (Spanish flu)** was the most significant pandemic of the 20th century, infecting nearly a third of the global population.
   - **Many diseases are zoonotic**, which means they are transmitted from animals to humans.
   - **Respiratory viruses**, including SARS-CoV-2, primarily spread when people breathe and inhale airborne particles (e.g., droplets, aerosols) containing the virus.
2. **Multi-cause frame:** The impact of the ongoing COVID-19 pandemic can still be felt across much of the globe. The pandemic has resulted in a large number of casualties, as well as significant socioeconomic disruption, ranging from unemployment to evictions to economic recession. Moreover, studies show that large-scale disease outbreaks will become increasingly frequent in the future. In order to minimize the impact of future pandemics, it is urgent to come up with an effective early response system.

   Multiple causes contribute to this complex issue. For example:
   - **The destruction of natural habitats** due to human activity (e.g., agriculture, logging) brings humans in close contact with wildlife, some of which carry diseases.
   - **Rising global temperatures** are causing some diseases to spread faster or expand their range.
   - **Livestock and wildlife markets** are not well-managed, creating favorable conditions for diseases to emerge, which could be passed on to humans.
   - **Globalization and the increase of human population** have allowed diseases to spread more quickly across borders.
3. **Multi-impact frame:** The impact of the ongoing COVID-19 pandemic can still be felt across much of the globe. The pandemic has resulted in a large number of casualties, as well as significant socioeconomic disruption, ranging from unemployment to evictions to economic recession. Moreover, studies show that large-scale disease outbreaks will become increasingly frequent in the future. In order to minimize the impact of future pandemics, it is urgent to come up with an effective early response system.

   Multiple impacts result from this complex issue. For example:
   - **Healthcare systems** are overwhelmed by pandemics, with many people unable to get the healthcare they need, and healthcare workers are overworked.
   - **Global supply chains** are heavily disrupted, particularly food supplies, which can lead to food shortages in some regions.
   - **Some industries**, such as tourism, aviation, and entertainment are hit hard by pandemics, and many people in these industries lose their jobs.
   - **Schools** have to close or shift to online teaching, severely affecting students, teachers, and parents.
4. **Multi-solution frame:** The impact of the ongoing COVID-19 pandemic can still be felt across much of the globe. The pandemic has resulted in a large number of casualties, as well as significant socioeconomic disruption, ranging from unemployment to evictions to economic recession. Moreover, studies show that large-scale disease outbreaks will become increasingly frequent in the future. In order to minimize the impact of future pandemics, it is urgent to come up with an effective early response system.

   Multiple solutions are needed to address this complex issue. For example:
   - **Early eradication** of a disease, which means removing a disease before it starts to spread out of control within a given region; this often involves placing restrictions on travel.
   - **Early suppression** of a disease, which means coexisting with the disease, but keeping daily cases under a certain threshold by promoting public health measures (e.g., using face masks, social distancing).
   - **Vaccine** development and mass immunization.
   - **Coordinated response** between different countries to prevent cross-border spread.

How severe a problem do you think early pandemic response is?

1=Not severe at all, 2=Slightly severe, 3=Somewhat severe, 4=Moderately severe, 5=Considerably severe, 6=Very severe, 7=Extremely severe

How urgent a problem do you think early pandemic response is?

1=Not urgent at all, 2=Slightly urgent, 3=Somewhat urgent, 4=Moderately urgent, 5=Considerably urgent, 6=Very urgent, 7=Extremely urgent

Please select which of the following you think is a good solution to early pandemic response.

1. Early eradication of the disease (remove a disease before it starts to spread out of control within a given region, involving placing restrictions on travel)
2. Early suppression of the disease (coexist with the disease, but keep daily cases under a certain threshold by promoting public health measures, e.g., using face masks, social distancing)
3. Multiple solutions are needed in addition to 1 and 2
4. Neither 1 nor 2
