# Supplementary material for: How does framing influence preference for multiple solutions to societal problems?
Source: PLoS One. 2023 May 17;18(5):e0285793. doi: 10.1371/journal.pone.0285793 (PMC10191302; doi:10.1371/journal.pone.0285793)
Supplement: S2 File — (DOCX) [file pone.0285793.s002.docx]

**Supporting Information**

**S2. Dichotomous Thinking Inventory** (Oshio, 2009)

Please indicate how much you agree with the following statements.

- All things work out better when likes and dislikes are clear.
- It works out best when even ambiguous things are made clear-cut.
- I dislike ambiguous attitudes.
- I want to clarify whether things are “good” or “bad.”
- I prefer it when boundaries are clear for all things.
- There are only “winners” and “losers” in this world.
- I think all people can be divided into “winners” or “losers.”
- People can clearly be distinguished as being “good” or “bad.”
- All questions have either a right answer or a wrong answer.
- I think of everyone as being either my friend or my enemy.
- I want to clearly distinguish what is safe and what is dangerous.
- Information should be defined as either true or false.
- I want to clarify whether things are beneficial to me or not.
- I prefer to classify information as being useful or useless for me.
- It is best when competitions have clear outcomes.

1= Strongly disagree, 2= Disagree, 3=Slightly disagree, 4= Slightly agree, 5=Agree, 6= Strongly agree
