# Supplementary material for: How does framing influence preference for multiple solutions to societal problems?
Source: PLoS One. 2023 May 17;18(5):e0285793. doi: 10.1371/journal.pone.0285793 (PMC10191302; doi:10.1371/journal.pone.0285793)
Supplement: S3 File — (DOCX) [file pone.0285793.s003.docx]

**Supporting Information**

**S3. Demographics**

Which gender do you identify with?

- Woman
- Man
- Non-binary
- Transgender
- Two spirited
- Other

What is your age (in years)?

[textbox for answer]

With which of the following do you identify? (select all that apply)

- White
- Black
- Indigenous peoples of North America
- Arab
- Latin, Central or South American
- Asian
- Other

Which of the following best describes your political views?

- Strongly liberal
- Liberal
- Slightly liberal
- Middle of the road
- Slightly conservative
- Conservative
- Strongly conservative

What is the highest level of education you have completed?

- Less than high school
- High school graduate
- Diploma/certificate
- Some college/university
- Bachelor's degree
- Graduate degree

What is your total annual household income before tax (in USD)?

- Less than $10,000
- $10,000 - $19,999
- $20,000 - $29,999
- $30,000 - $39,999
- $40,000 - $49,999
- $50,000 - $59,999
- $60,000 - $69,999
- $70,000 - $79,999
- $80,000 - $89,999
- $90,000 - $99,999
- $100,000 - $109,999
- $109,999 - $119,999
- $119,999 - $129,999
- $129,999 - $139,999
- $139,999 - $149,999
- More than $150,000

How many people are there in your household (including yourself)?

[textbox for answer]
