# Supplementary material for: How does framing influence preference for multiple solutions to societal problems?
Source: PLoS One. 2023 May 17;18(5):e0285793. doi: 10.1371/journal.pone.0285793 (PMC10191302; doi:10.1371/journal.pone.0285793)

**Supporting Information**

**S4. Additional results**

**Table S1.** Results of the one-way ANCOVA for preference for multi-solutions, with gender, age, ethnicity, political orientation, education level, and annual personal income as covariates.

|  | **df** | **SS** | **MS** | **F value** | ***p*** |
| --- | --- | --- | --- | --- | --- |
| Condition | 3 | 37 | 12.32 | 1.70 | 0.165 |
| Gender | 1 | 114 | 114.29 | 15.80 | <.001 |
| Age | 1 | 35 | 34.56 | 4.78 | 0.03 |
| Ethnicity | 1 | 80 | 80.40 | 11.11 | <.001 |
| Political orientation | 1 | 207 | 206.77 | 28.58 | <.001 |
| Education level | 1 | 235 | 234.65 | 32.44 | <.001 |
| Annual personal income | 1 | 92 | 92.45 | 12.78 | <.001 |
| Residuals | 1356 | 9809 | 7.23 |  |  |

**Table S2.** Results of the one-way ANCOVA for average perceived severity, with gender, age, ethnicity, political orientation, education level, and annual personal income as covariates.

|  | **df** | **SS** | **MS** | **F value** | ***p*** |
| --- | --- | --- | --- | --- | --- |
| Condition | 3 | 1.7 | 0.57 | 0.42 | 0.74 |
| Gender | 1 | 12.4 | 12.38 | 9.18 | 0.002 |
| Age | 1 | 2.4 | 2.37 | 1.76 | 0.19 |
| Ethnicity | 1 | 24.5 | 24.55 | 18.20 | <.001 |
| Political orientation | 1 | 20.9 | 20.93 | 15.52 | <.001 |
| Education level | 1 | 0.1 | 0.08 | 0.06 | 0.81 |
| Annual personal income | 1 | 0.9 | 0.94 | 0.70 | 0.40 |
| Residuals | 1356 | 1829.2 | 1.35 |  |  |

**Table S3.** Results of the one-way ANCOVA for average perceived urgency, with gender, age, ethnicity, political orientation, education level, and annual personal income as covariates.

|  | **df** | **SS** | **MS** | **F value** | ***p*** |
| --- | --- | --- | --- | --- | --- |
| Condition | 3 | 3.6 | 1.20 | 0.91 | 0.43 |
| Gender | 1 | 10.3 | 10.29 | 7.84 | 0.005 |
| Age | 1 | 1.3 | 1.29 | 0.99 | 0.32 |
| Ethnicity | 1 | 21.8 | 21.78 | 16.59 | <.001 |
| Political orientation | 1 | 30.3 | 30.27 | 23.07 | <.001 |
| Education level | 1 | 1.7 | 1.68 | 1.28 | 0.26 |
| Annual personal income | 1 | 0.6 | 0.65 | 0.49 | 0.48 |
| Residuals | 1356 | 1779.7 | 1.31 |  |  |

**Table S4.** Chi-square test results for the number of participants who chose each option for each of the eight problems in each condition.

| **Problem** | **χ2** | **df** | ***p*** |
| --- | --- | --- | --- |
| Climate change | 6.97 | 9 | 0.64 |
| Crop yield and biodiversity | 8.77 | 9 | 0.46 |
| Food waste | 10.44 | 9 | 0.32 |
| Plastic pollution | 8.48 | 9 | 0.49 |
| Homelessness | 5.77 | 9 | 0.76 |
| Police reform | 10.86 | 9 | 0.29 |
| Public education | 11.40 | 9 | 0.25 |
| Early pandemic response | 8.37 | 9 | 0.50 |

**Table S5.** Correlations between preference for multiple solutions and other variables (perceived severity, perceived urgency, level of dichotomous thinking, demographic variables), by condition.

| **Preference for multiple solutions** | **Control** | | **Multi-cause** | | **Multi-impact** | | **Multi-solution** | |  |  |  |
| --- | --- | --- | --- | --- | --- | --- | --- | --- | --- | --- | --- |
|  | ***r*** | ***p*** | ***r*** | ***p*** | ***r*** | ***p*** | ***r*** | ***p*** |  |  |  |
| Perceived severity | 0.25*** | <0.001 | 0.21*** | <0.001 | 0.16*** | <0.001 | 0.29*** | <0.001 |  |  |  |
| Perceived urgency | 0.28*** | <0.001 | 0.24*** | <0.001 | 0.18*** | <0.001 | 0.29*** | <0.001 |  |  |  |
| Dichotomous thinking | −0.10** | 0.001 | −0.12** | 0.002 | −0.13*** | <0.001 | −0.12** | 0.002 |  |  |  |
| Gender | −0.08 | 0.1 | −0.12* | 0.011 | −0.05 | 0.24 | −0.11* | 0.015 |  |  |  |
| Age | 0.03 | 0.44 | 0.07 | 0.07 | 0.07 | 0.05 | 0.03 | 0.5 |  |  |  |
| Ethnicity | 0.1* | 0.03 | 0.05 | 0.31 | 0.07 | 0.14 | 0.06 | 0.17 |  |  |  |
| Political orientation | −0.16*** | <0.001 | −0.07 | 0.08 | −0.15*** | <0.001 | −0.1* | 0.014 |  |  |  |
| Education level | −0.04 | 0.31 | −0.11* | 0.01 | −0.17*** | <0.001 | −0.14*** | <0.001 |  |  |  |
| Personal annual income | 0.005 | 0.89 | 0.03 | 0.38 | −0.04 | 0.28 | 0.002 | 0.97 |  |  |  |

**Table S6.** Results of multiple regression GLM. Dependent variable is preference for multiple solutions. Independent variables include perceived severity, perceived urgency, dichotomous thinking, and demographic variables. Interactions between preference for multiple solutions and perceived severity, perceived urgency, dichotomous thinking are included.

| **Coefficients** | **Estimate** | **Std. Error** | **z value** | **Pr(>\|z\|)** |
| --- | --- | --- | --- | --- |
| Intercept | −0.40 | 0.79 | −0.50 | 0.62 |
| Multi-cause | 0.51 | 0.96 | 0.53 | 0.60 |
| Multi-impact | 1.17 | 0.98 | 1.20 | 0.23 |
| Multi-solution | 0.21 | 1.03 | 0.20 | 0.84 |
| Perceived severity | −0.12 | 0.24 | −0.50 | 0.62 |
| Perceived urgency | 0.64 | 0.26 | 2.51 | 0.01 |
| Dichotomous thinking | −0.27 | 0.14 | −1.92 | 0.05 |
| Gender | −0.16 | 0.12 | −1.37 | 0.17 |
| Age | 0.01 | 0.00 | 1.64 | 0.10 |
| Ethnicity | 0.12 | 0.15 | 0.76 | 0.45 |
| Political orientation | −0.06 | 0.03 | −1.92 | 0.06 |
| Education level | −0.20 | 0.06 | −3.23 | 0.001 |
| Personal annual income | 0.00 | 0.00 | 1.43 | 0.15 |
| Multi-cause: Perceived severity | −0.22 | 0.35 | −0.63 | 0.53 |
| Multi-impact: Perceived severity | 0.18 | 0.34 | 0.54 | 0.59 |
| Multi-solution: Perceived severity | 0.35 | 0.37 | 0.95 | 0.34 |
| Multi-cause: Perceived urgency | 0.12 | 0.36 | 0.34 | 0.73 |
| Multi-impact: Perceived urgency | −0.39 | 0.35 | −1.12 | 0.26 |
| Multi-solution: Perceived urgency | −0.35 | 0.38 | −0.91 | 0.36 |
| Multi-cause: Dichotomous thinking | −0.04 | 0.19 | −0.19 | 0.85 |
| Multi-impact: Dichotomous thinking | −0.06 | 0.19 | −0.34 | 0.74 |
| Multi-solution: Dichotomous thinking | −0.05 | 0.20 | −0.24 | 0.81 |

The formula of the GLM is as follows:

$$Preference for multiple solution \sim Condition*Perceived severity+$$

$$Condition*Perceived urgency+$$

$$Condition*Dichotomous thinking+$$

$$Gender+Age+Ethnicity+$$

$$Political orientation+Education level+$$

$$Personal annual income$$

**Figure S1.** Percentage of participants who chose each option for each of the eight problems in each condition.


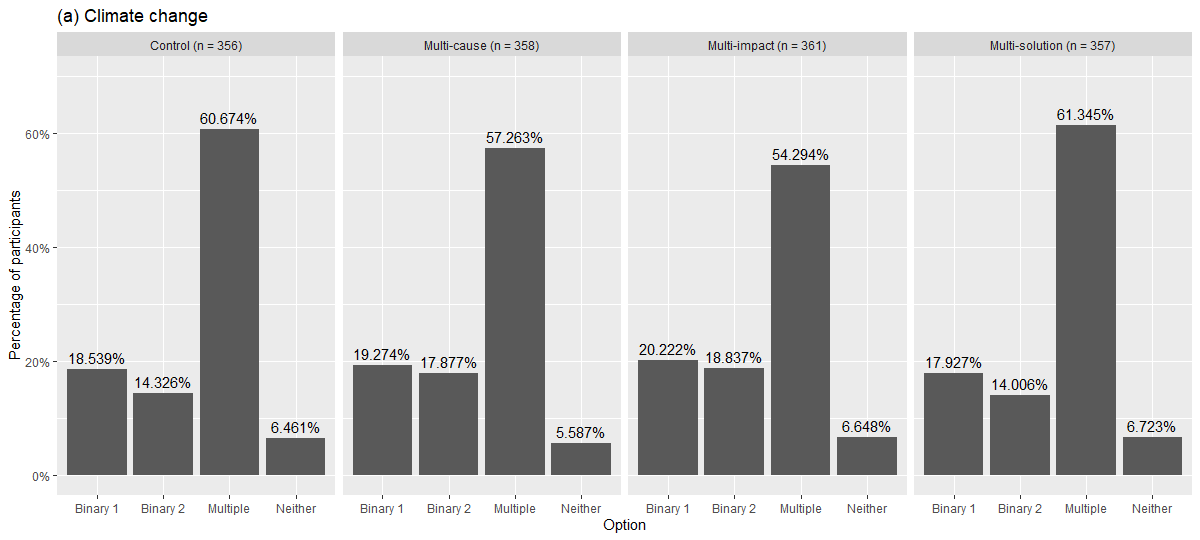


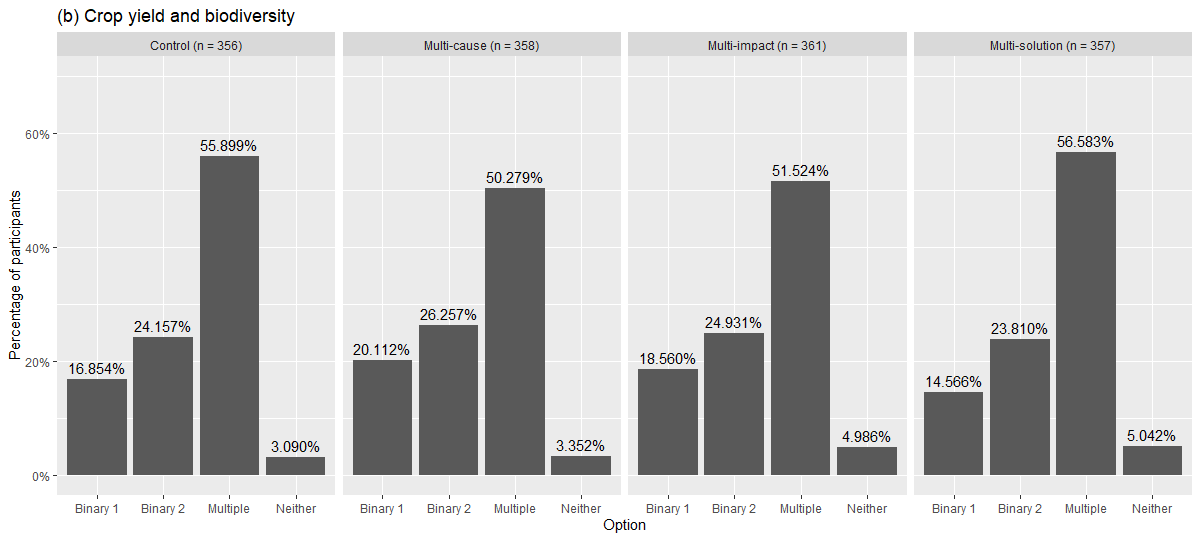


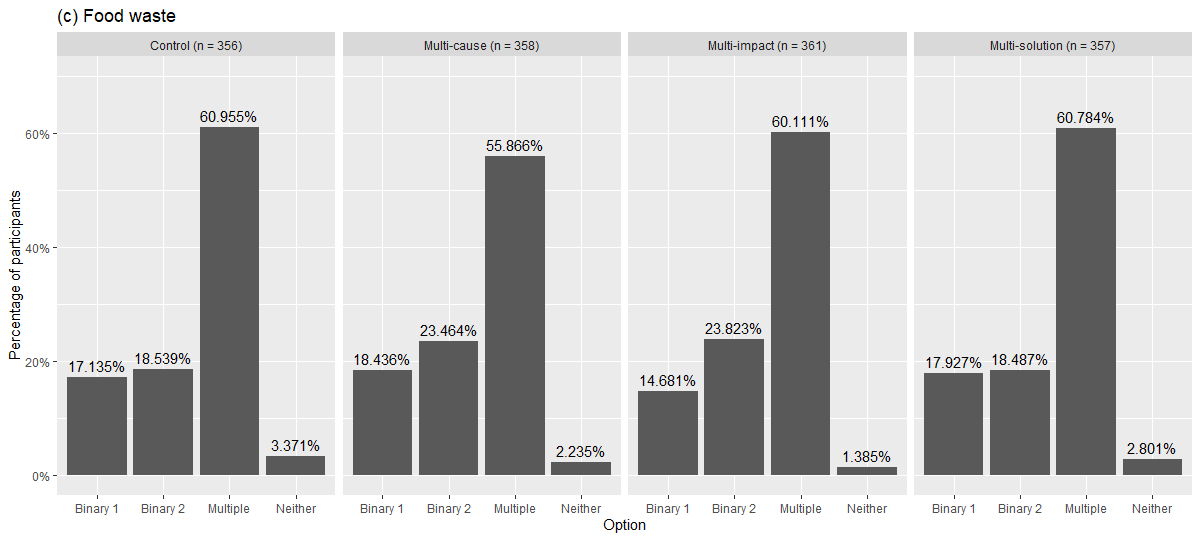


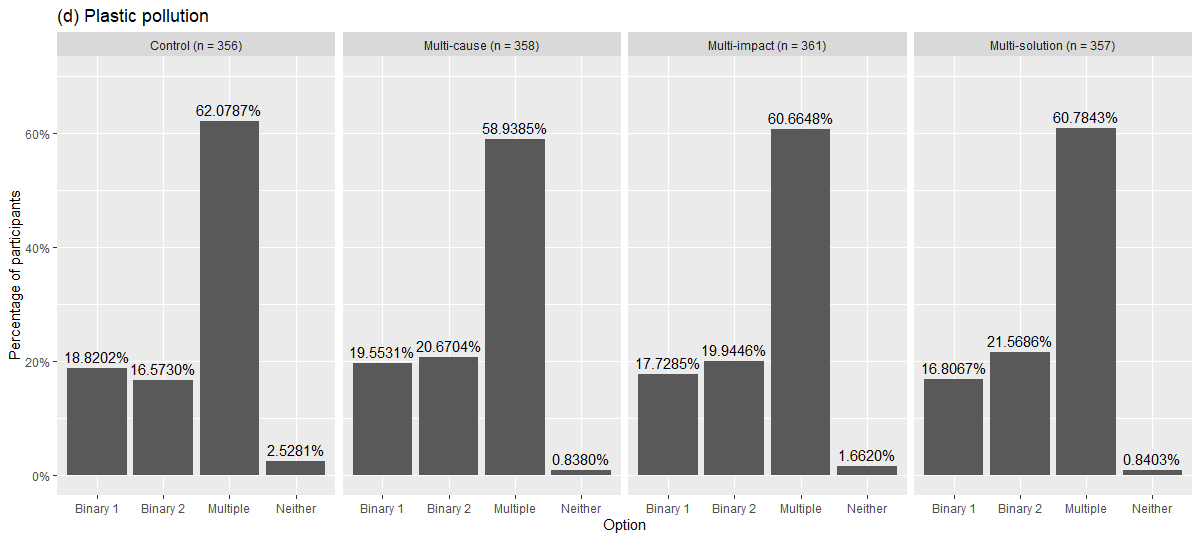


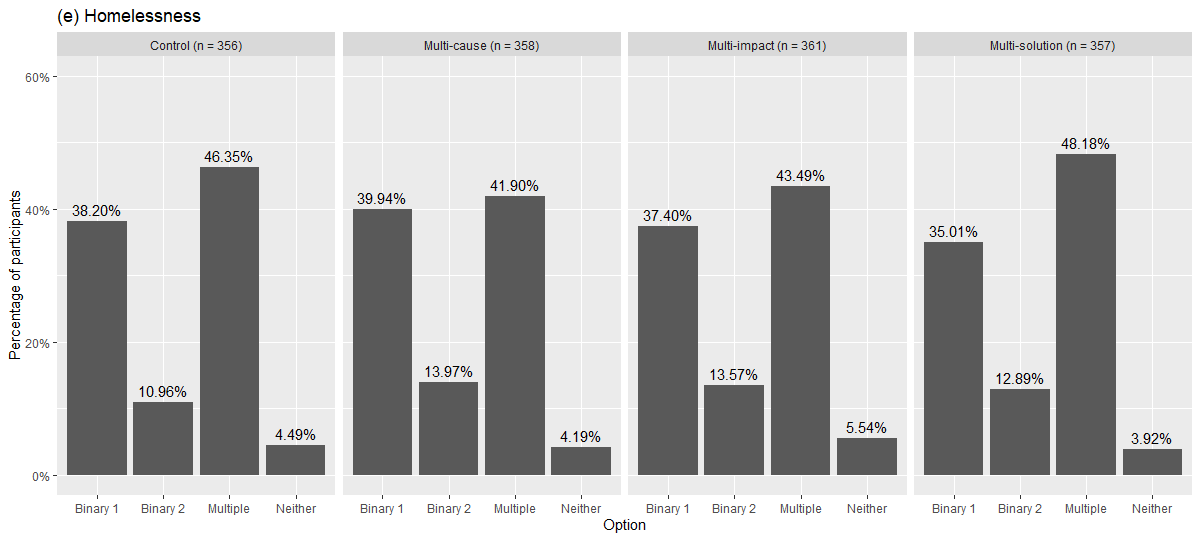


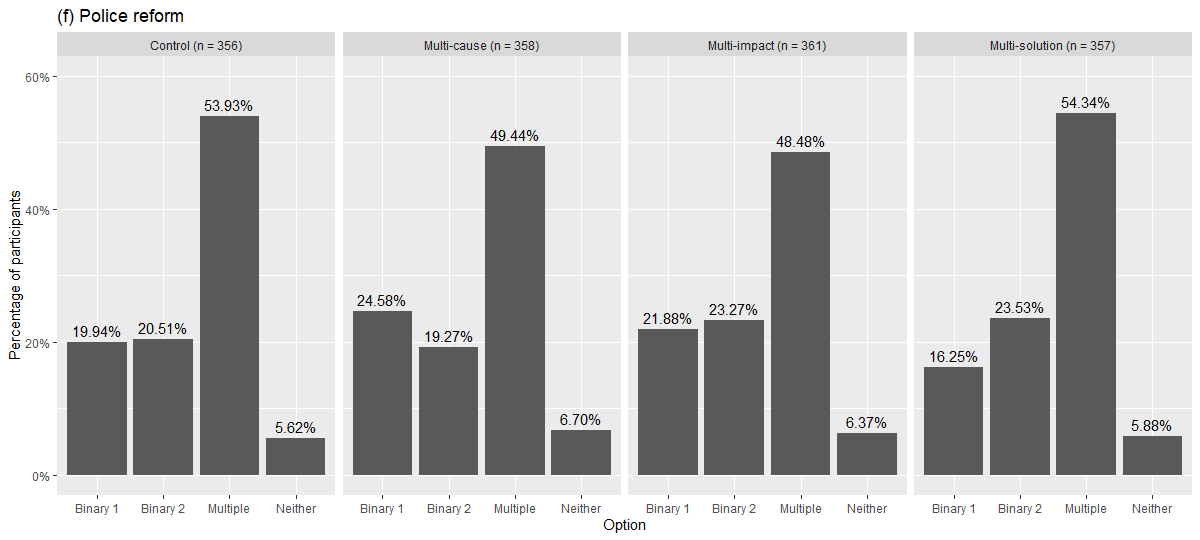


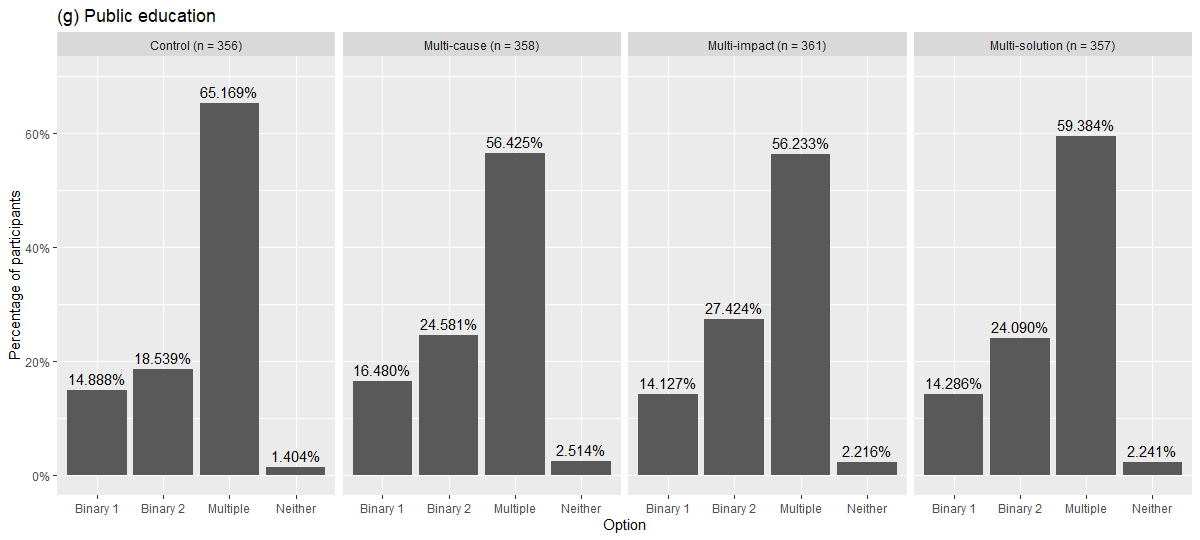


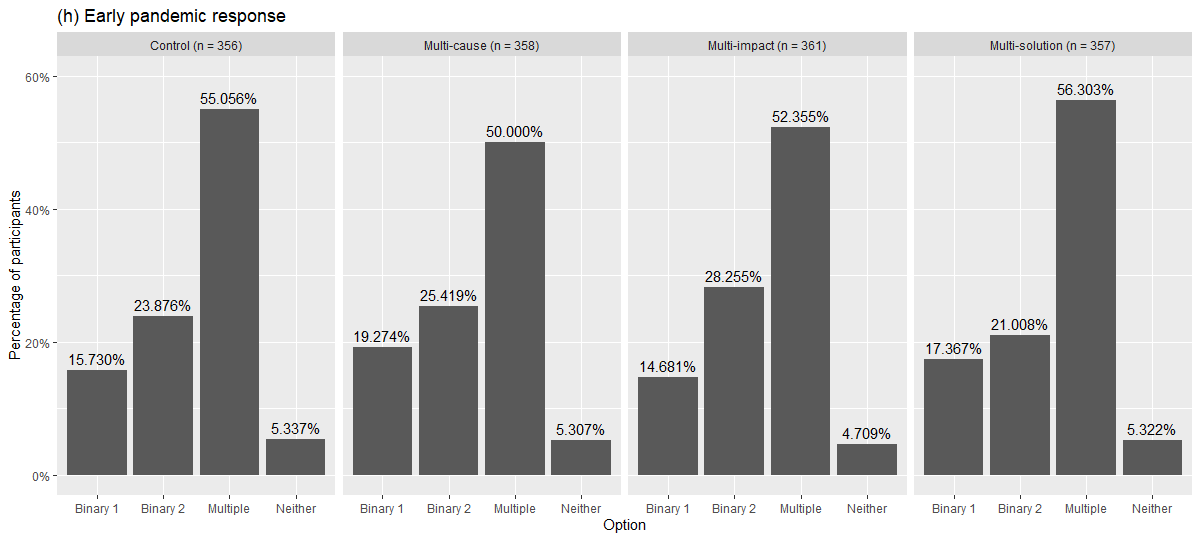


**Figure S2.** Percentage of participants that chose each option in temporal sequence (disregarding problem), for each condition.


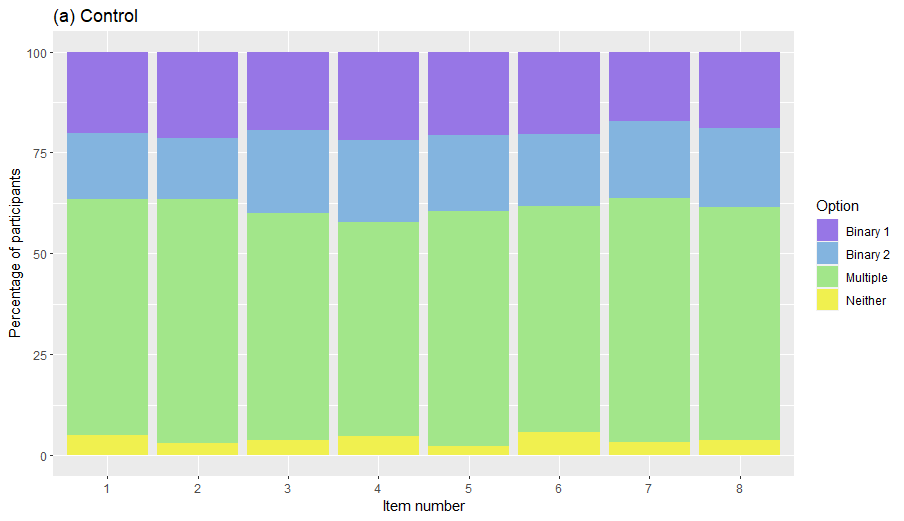


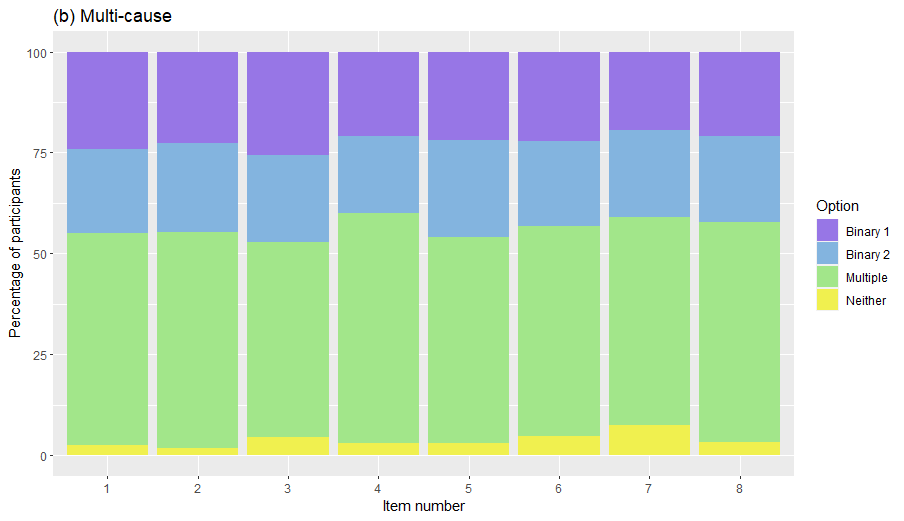


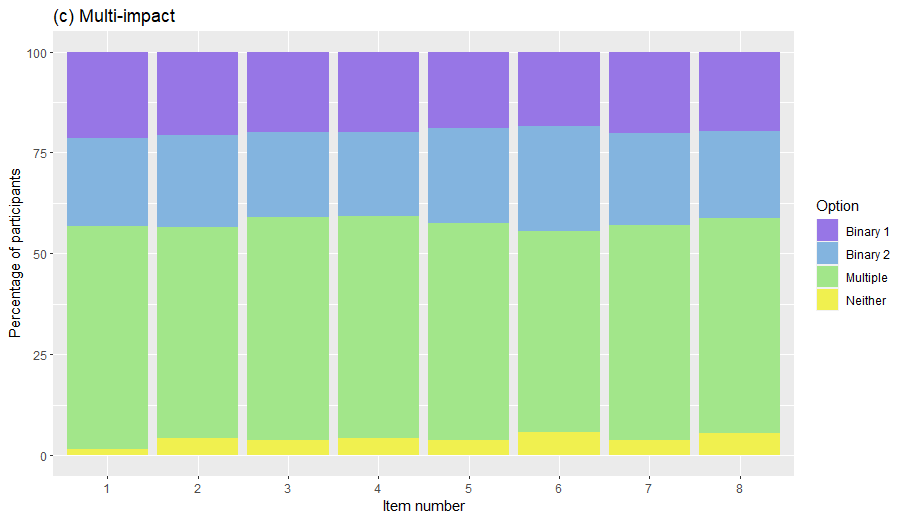


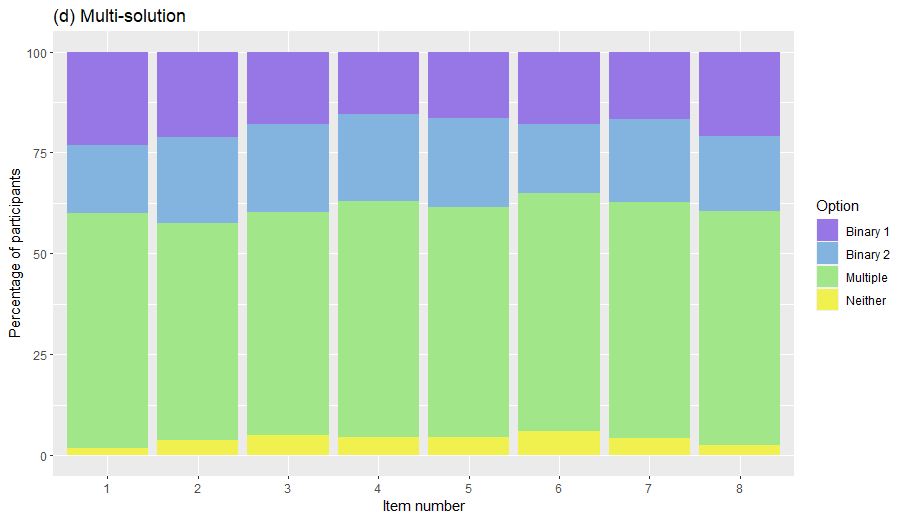


**Figure S3.** Scatterplots for correlations between political orientation and preference for multiple solutions (a–d), and between education level and preference for multiple solutions (e–h).


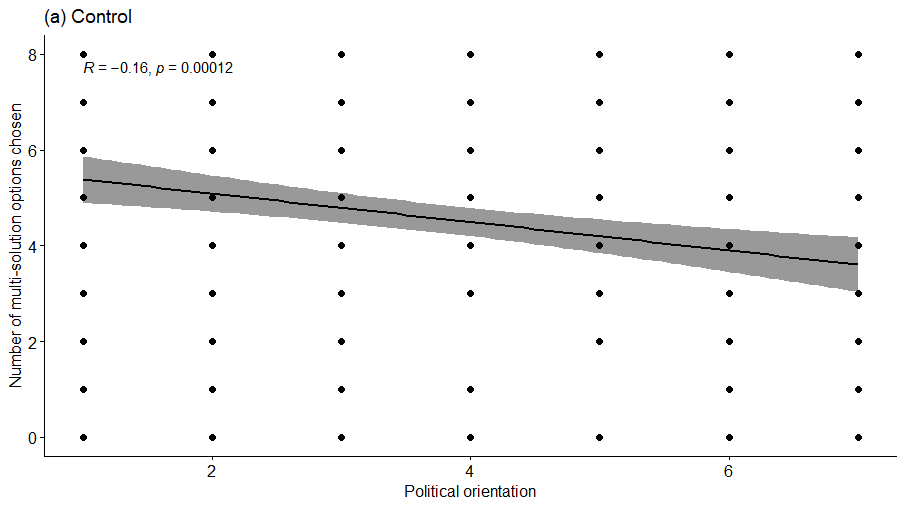


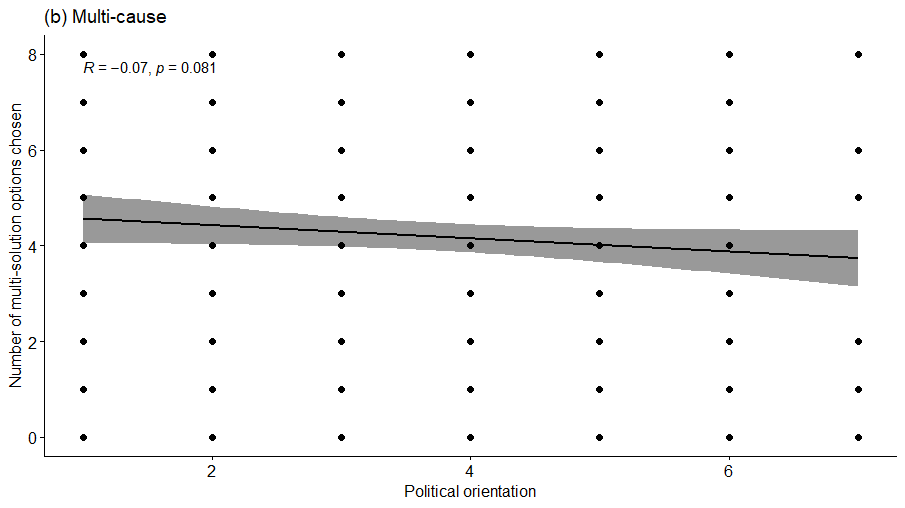


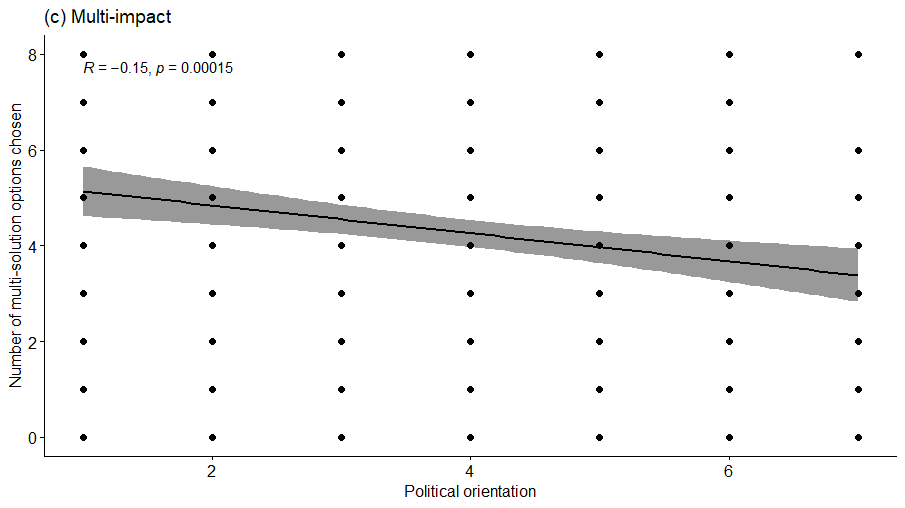


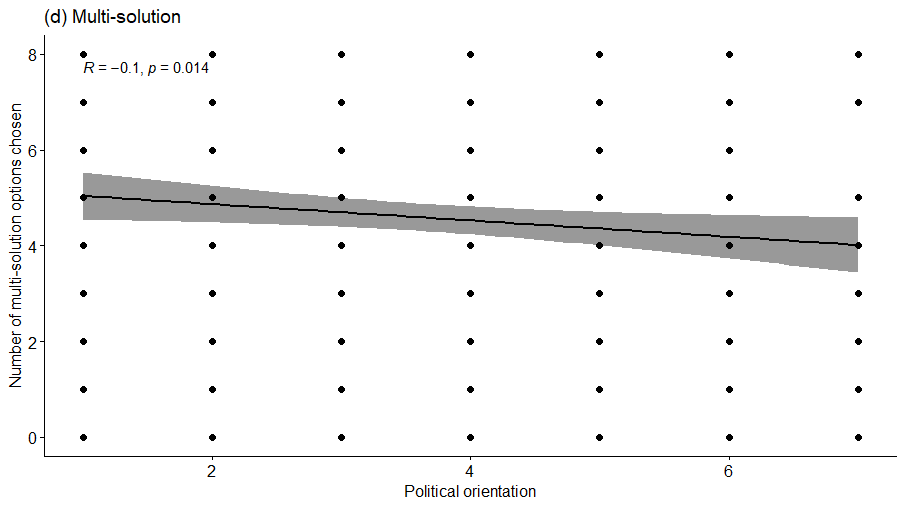


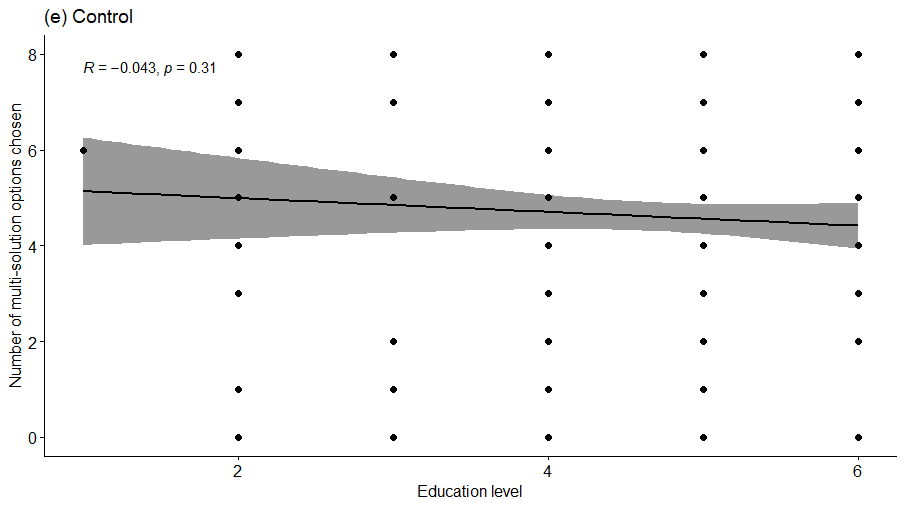


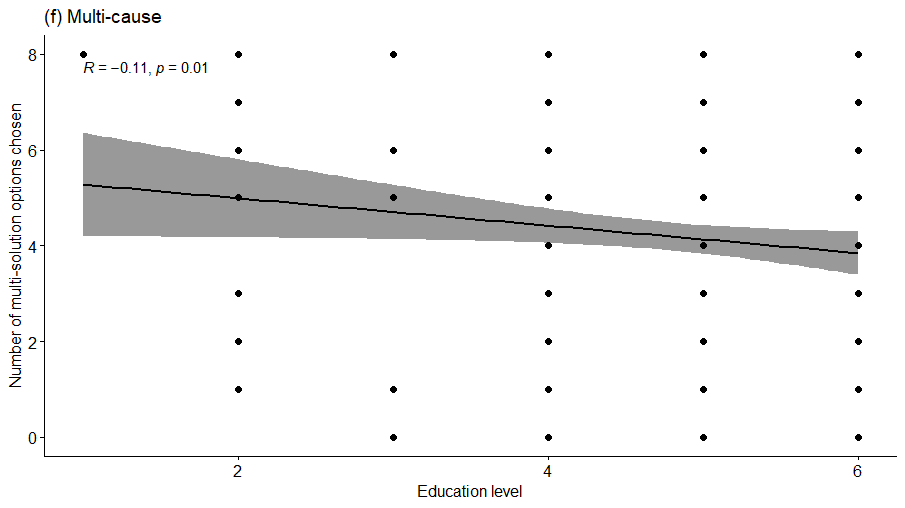


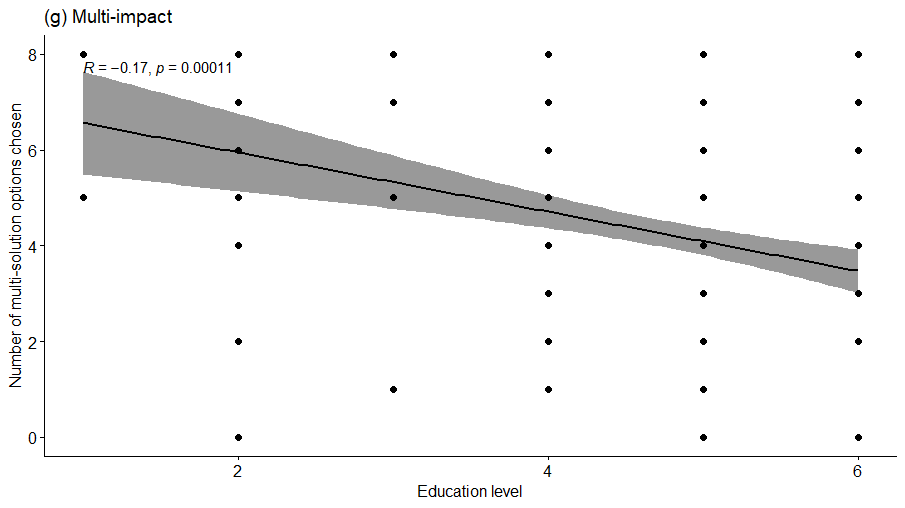


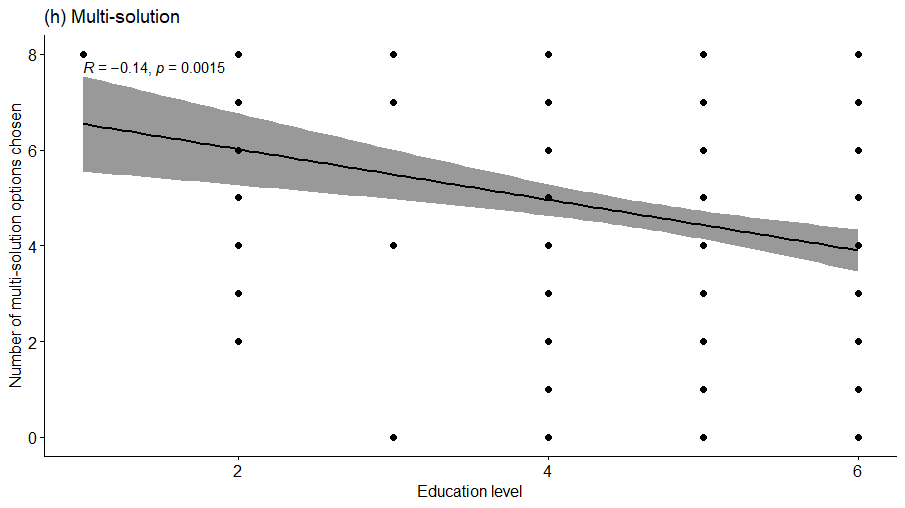

Supplement: S4 File — (DOCX) [file pone.0285793.s004.docx]
