# Supplementary material for: How does framing influence preference for multiple solutions to societal problems?
Source: PLoS One. 2023 May 17;18(5):e0285793. doi: 10.1371/journal.pone.0285793 (PMC10191302; doi:10.1371/journal.pone.0285793)
Supplement: Attachment — Submitted filename: Response to Reviewers.docx [file pone.0285793.s005.docx]

**Response to Reviewers**

Reviewer #1: This study focuses on an important question, using an extensive sample, with preregistered research methods. The findings are important. I have several comments focusing especially on interpretation and the request for additional presentation of results.

(1) Interpretation A: Might the limited effect of framing compared with the control condition be partially attributed to the strong "manipulation check" that was used, where participants were required to place, in order, all of the presented bullet points per item. It is possible that this requirement may have elicited quite sustained and deep processing from all participants, in turn, encouraging a multi-solution perspective, and perhaps particularly so across the multiple items.

- Thank you for raising this point. However, we believe that the manipulation check in the three experimental conditions did not have a major effect on encouraging participants to choose the multi-solution option. This is because participants assigned to the control condition, which did not have the manipulation check, also largely preferred the multi-solution option. This indicates that the baseline for multi-solution preference was high. We have expanded our discussion to explain why this might have been the case (p.14).

(2) Interpretation B: An additional potential reason for participants to choose the "multiple" solutions option (phrased as "Multiple solutions are needed in addition to 1 and 2") is that it is shorter than either the first or second options and could be chosen with a quick reading of the first and second options; if the first and second (longer) options seemed at least plausible on a quick reading, then choosing "both" involves less cognitive effort than carefully reading and differentiating between the first and second (longer) options. Thus part of the effects may be attributed to a tendency to prefer efficiency of decisions by participants. The "multiple solutions" response is also the same option across all of the items, and so (again) requires less careful reading/consideration.

- This is a great critique of our survey design and might explain the high preference for the multi-solution option. We have added this to the discussion (p.14).

Related to this: An exploratory analysis might examine if the same patterns are observed for only the first few items that participants responded to. Given that the order of stimulus presentation was randomized, this could be informative with regard to a fatigue or cognitive efficiency account.

- We have included figures showing the percentage of participants that choose each option in temporal sequence in the Supplementary Information (Figure S2). The results showed a consistently high preference for multiple solutions throughout the duration of the experiment (p.12).

(3) Analyses: Setting aside the number of participants who chose the multiple solutions option, I wonder if some (exploratory) suggestions might be derived from comparing the frequency of the choices for 1 vs. 2 for the different topics. For instance, whereas there seem to be generally approximately equal number of participants who chose option 1 vs. chose option 2 for the majority of topics, there is a marked difference for the topic of homelessness, with the proportion chosing binary option 1 nearly as high as those choosing multiple solutions. Also, given how different the responses to the homelessness topic are from the other topics, it might be worthwhile examining patterns excluding this topic.

- Thank you for bringing up this interesting point, we have expanded upon this in the discussion (p.16). We think that communication with the general public plays an important role in addressing some of the patterns we have observed for choices 1 and 2. This is especially relevant to the solutions to homelessness, as many people tend to have misconceptions on direct cash transfers (even though some studies have demonstrated that they are an effective way of reducing homelessness), which probably explains a lot more participants choose option 1 (social services) than option 2 (cash transfers) for homelessness. We believe that a multi-pronged approach is necessary: improving the education system to better educate people on solutions to important issues; better scientific communication, especially regarding solutions that can be unintuitive to the average person; reducing political polarization, since certain types of solutions tend to be disregarded due to political reasons, regardless of their efficacy.
- We have conducted analyses excluding the problem of homelessness, but did not find any major differences in the overall patterns. We think this is not surprising, as despite the unusual patterns in options 1 and 2, the multi-solution option was still the most frequently chosen option across all conditions in regards to homelessness.

(4) In the Introduction, with respect to framing in relation to psychological distance, it is notable that there are questions about the extent to which evidence in favor of construal level theory may be the outcome of publication bias; see, especially https://psyarxiv.com/r8nyu/

- Thank you for providing this enlightening study. We are aware that there are problems with framing using psychological distance, and have incorporated this point into the discussion (p.15).
- We have expanded the introduction to provide a better justification for the frames used in the current study (p.4).

(5) The inverse relation between higher levels of education and choosing the multi-solution option seems puzzling, and one wonders if there are other possible accounts. The descriptive statistics for the demographic measures are not provided and should be given. Additionally, scatterplots of the inverse correlations should be examined and provided (e.g., in the supplementary material) to assess to what degree the (small) negative correlations are representative.

- We have expanded the discussion on the relationship between education level and preference for multiple solutions (p.16).
- We have included scatterplots for the correlations involving demographic variables (Figure S3).
- The descriptive statistics for the demographic measures are provided in the materials and methods section (p.6), but please let us know if we have misunderstood what you are asking.

Specific Comments
- The manuscript does not have page numbers; please add.
- Introduction, first page, word missing, text should read, "allows humans to make decisions"
- Discussion, text amiss: "in some study, participants with higher..." (should read "studies"?)

- Thank you for catching these typos. We have fixed all of them.

Reviewer #2: Summary: This between-subjects experiment was designed to test whether describing real-world problems in different ways would have an influence on what people would choose as a "good solution" to the problem as well as how severe and how urgent they think the problem is. More specifically, the authors were interested in whether their different descriptions would result in participants being more likely to choose the option of "multiple solutions" as a "good solution" (and, potentially, less likely to choose only one specific solution as a "good solution." This is because their manipulation involved describing the problem and then listing multiple causes, multiple impacts, or multiple problems (three experimental conditions) as compared to listing multiple facts about the problem (one control condition). There were no significant differences found in the choices made by participants based on these differences in description. The impact of certain person variables--such as dichotomous thinking, political affiliation, and highest level of education--on these decisions were also explored. Interesting relationships were observed in exploratory analyses, such as the positive correlations between the likelihood of choosing the option of "multiple solutions" and perceiving the problem as more severe and more urgent and the negative correlation between the likelihood of choosing the option of "multiple solutions" and dichotomous thinking. My overall impression was generally positive. They make a strong argument for the importance of conducting more research on the topic of dichotomous thinking outside of clinical contexts, but I think there are some limitations that the authors need to address.

Major Issues:

1) The authors state at the beginning of their discussion section that their manipulation "had little impact" on the choices that people made. I understand that this is a common expression that should be interpreted as "had no demonstrable impact;" however, I think the latter more clearly describes the findings of this experiment. In the abstract, the authors state "these findings show a limited impact of framing on multi-solution preferences," and I find this even more misleading. The phrase "limited impact" is also used in their conclusion. None of the evidence presented suggests that the manipulation played a significant role in participants' decisions. I think this needs to be clearly summarized. At the same time, I acknowledge that the limitations of the study might have prevented the researchers from finding an effect that might be evidence under different conditions; however, that does not change their current results.

- Thank you for this critique. We have revised the wording to more accurately reflect our results, which indeed showed no obvious impact of framing of preference for multiple solutions.

2) I noted a potential limitation that was not addressed by the authors. The author might not have found the effect they were interested in because they did not explicitly ask participants to consider the information provided when answering questions about the severity of the problem, the urgency of the problem, or their preferred solution. Without such an instruction participants may have relied more heavily on their pre-existing beliefs, which might help explain why some of the person variable were good predictors of whether people would choose the option of "multiple solutions."

- This is a great point. We have added this to the discussion (p.17).

Minor Issues:

1) I had an issue with the question that was used to ask participants about their preferred solution. The question asks the participants to select a "good solution;" however, I was not clear on why the question was not about the "best solution" and whether such a change might have made a difference.

- Our rationale for using the term “good solution” was because we thought “best solution” sounded more exclusive and could prime participants to think more dichotomously. However, in some of our pilot studies, we did use the term “best solution”, but the results were not sufficiently different from the current study.

2) I could not tell which style guide was being used for the reference section. There were some inconsistencies in how names were formatted and whether periods were or were not used following authors' initials.

- Thank you for catching this. We have corrected the formatting of the reference section according to journal guidelines.
